# Pakistan’s national COVID-19 response: lessons from an emergent response to the pandemic

**DOI:** 10.3389/fpubh.2024.1379867

**Published:** 2024-08-05

**Authors:** Adnan Ahmad Khan, Mujahid Abdullah, Romesa Khan, Twangar Kazmi, Faisal Sultan, Shirin Aamir, Aamir Ashraf Khawaja, Ayesha Khan

**Affiliations:** ^1^Research and Development Solutions (RADS), Islamabad, Pakistan; ^2^Ministry of National Health Services, Regulations and Coordination, Islamabad, Pakistan; ^3^Akhter Hameed Khan Foundation, Islamabad, Pakistan; ^4^Shaukat Khanum Memorial Cancer Hospital and Research Center, Lahore, Pakistan; ^5^Independent Consultant, Islamabad, Pakistan

**Keywords:** COVID-19, Pakistan, NCOC, multisectoral collaboration, data-driven intervention, public health policy, resource-constrained settings

## Abstract

**Introduction:**

In 2020, Pakistan faced the formidable challenge of the COVID-19 pandemic with an existing yet disjointed healthcare infrastructure, that included by over 18,000 public and an estimated 75,000 private health facilities and some elements of an epidemic surveillance and response system. This descriptive study examines how Pakistan developed a COVID-19 response, driven by establishing a central coordination and decision-making mechanism to overcome these systemic challenges.

**Methods:**

The study is based on interviews and interactions of the many actors in the response by the authors, who also participated in nearly all proceedings of the National Command and Operation Centre (NCOC) and many of the National Coordination Committee (NCC). This information is supplemented by reviewing documents, reports, news items, media and social media, and journal articles.

**Results:**

The study highlights the critical role of political arrangement, where the NCC, comprising of ministers, bureaucrats, and military personnel, facilitated federal and provincial integration. The NCC found resources and set policy. Its direction was implemented by the NCOC, a top-down yet inclusive platform, integrated political, military, and civil society actors, to ensure cohesive decision-making and implementation. It provided technical guidance, harnessed data for strategic decisions and held implementers accountable. At its peak, the NCOC boasted nearly 300 personnel, including high-ranking military officers, a stark contrast to the limited staffing in most ministries. In addition, the response’s success is attributed to the perception of COVID-19 as an existential threat, leading to unprecedented collaboration and decisive actions that were enforced authoritatively.

**Conclusion:**

Pakistan’s experience offers valuable insights for proactive management of health emergencies in resource-limited settings. It underscores the necessity for inter-sectoral dialog and data-driven policy implementation, especially in the context of political economies where activity-driven governance often overshadows objective-driven policy execution. However, the lessons from the COVID-19 response, including a blueprint for future epidemic responses and lessons for use of data and evidence in developing country health systems, if not institutionalized, risk being lost in the post-pandemic era.

## 1 Introduction

Crisis, resilience, and innovation interwove intricately in the response to SARS-CoV-2 epidemic by Pakistan (1, 2). Pakistan has a wide health infrastructure with many public sector resources that are not always coordinated (1, 3, 4). The coordination is further challenged under the 18th amendment to the constitution which devolved the responsibility for health management to provinces independently. Thus, although many necessary pieces to fight the epidemic were present, they were not necessarily best placed to do so, and many others had to be developed. Foremost a coordination mechanism was needed to synchronize a massive multisectoral response comprising all aspects of the society. The effectiveness of this response is evident from the fewer case transmission and deaths due to COVID-19 in Pakistan, compared to its regional neighbors (2, 5). We explore how this response unfolded and what lessons may be learnt from it.

The initial lack of knowledge of the novel biology and transmission potential of COVID-19 caught governments all over the world by surprise and led to widely varying health system responses in different countries. For instance, the Chinese government responded with centralized control, devoted considerable resources to medical facilities and initiated sweeping lockdowns (6, 7). Other Asian economies like Singapore, Hong Kong, and South Korea also relied on COVID-19 testing, isolation and quarantine. Some European countries such as Italy and Spain, at least initially, hesitated to lockdown and delayed containment measures. The United States with its federal structure of governance, saw diverse responses that ranged from very strict lockdowns, school and business closures and stringent masking to very open or even *laissez-faire* approaches. While some of this variation was in response to different types of the epidemic between US states, at least some of it was driven politically (8). These myriad variations in response served as natural experiments that over time provided information about alternate scenarios, responses and improvisations.

Countries applied lessons from previous experiences with outbreaks to their COVID-19 management (9). Many mounted centralized responses (9, 10), acted quickly (11, 12), and devoted substantial public funds up front. Pakistan spends relatively little in health, has a high burden of communicable diseases (2), and few systems to combat them, save perhaps an elaborate system to identify polio cases. In managing the epidemic, Pakistan had to manage the surging cases, and deaths while counterbalancing extensive economic and social disruption from lockdowns (13).

This paper attempts to document the national COVID-19 response of the Government of Pakistan. It describes central coordination by the federal government, its political cooperation with the legally devolved provinces to coopt and build upon existing structures, while creating new ones that were needed. Our goal is to look at Pakistan as a case study of managing pandemics in developing countries.

## 2 Methodology

This descriptive paper outlines how Pakistan’s COVID-19 response evolved, by describing what systems existed, what additions were made, and the processes of the response. The information about administrative structures of the relevant institutions and stakeholders, as well as about the policy response to disease containment, comes from attending the daily official meetings of the National Command and Operation Center (NCOC), regular meetings of the National Coordination Committee (NCC), which was formed to respond to the pandemic, and personal meetings with officials of Ministry of National Health Services, Regulations and Coordination (MoNHSRC), National Institute of Health (NIH), National Emergency Operation Centre (14), and the National Database and Registration Authority (NADRA) who participated in aspects of the national COVID-19 response. We also used government and other reports, journal articles, newspapers and media sources where needed.

## 3 Pakistan’s COVID-19 response

### 3.1 Overview of the epidemic

Until June 2022, five waves of COVID-19 had affected Pakistan (Table 1), based on criteria for determining the start and end dates of every wave from existing COVID-19 literature^1^ (12).

**Table 1:** Dates for COVID-19 waves in Pakistan.

Wave	Start date	End date	Duration (days)
Wave 1	3rd April 2020	31st August 2020	150
Wave 2	12th October 2020	16th February 2021	128
Wave 3	23rd February 2021	22nd June 2021	120
Wave 4	6th July 2021	29th November 2021	147
Wave 5	7th December 2021	23rd February 2022	83

### 3.2 Institutional arrangements

Pakistan has a federal parliamentary republic with four provinces and three jurisdictions. Each province pursues its own health policy, provides services, and responds to public health emergencies at its own discretion. The federal Ministry of National Health Services, Regulations and Coordination (MoNHSRC) coordinates, provides guidance and reports health data between the provinces and internationally. For the COVID-19 pandemic, this decentralization meant that each province potentially had to learn and act on its own, with likelihood of breakdowns in coordination due to political expediencies.

**Figure 1:**
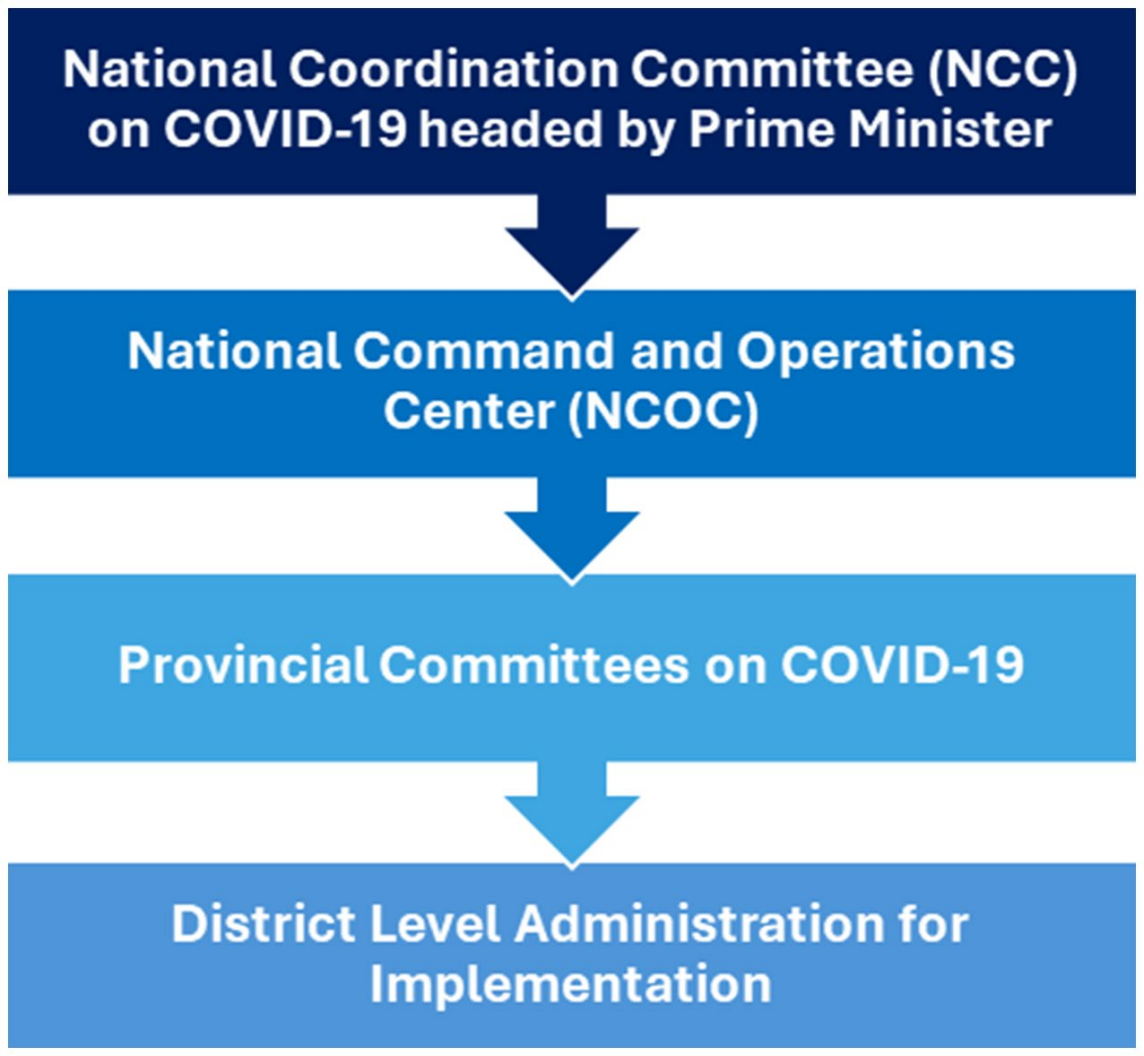
Governance structure of the NCOC.

On 13^th^ March 2020, a National Coordination Committee (NCC) was established to respond to COVID-19. It was chaired by the Prime Minister and included the army chief, some senior army generals, provincial chief ministers, and all federal ministers. This committee met weekly to decide policy and ensure political coordination between provinces. This policy guidance was translated into action by a National Command and Operation Center (NCOC), constituted in April 2020. It was co-chaired by the Minister of Planning. The Director General of NCOC was a senior military official with the rank of a general. The health ministry provided technical support. It also invited provincial chief ministers weekly and other ministries as needed. NCOC operations were funded by the Federal Government through the health ministry. The NCOC met daily. Its directions were carried out by provincial committees on COVID19, assisted at the district level through the civil set up headed by the Deputy Commissioner. The health and planning ministers reported progress to the Prime Minister and the cabinet, as part of the NCC (Figure 1).

In daily meetings, the NCOC reviewed data including COVID-19 cases, deaths, hospitalizations, testing, reports of compliance with non-pharmaceutical interventions, global trends, and other related news (15) and any other relevant information. These were translated into instructions to provincial implementers (16). The day-to-day operations on the NCOC decisions were almost exclusively carried out by over 300 army personnel deputed full time to the NCOC. At the beginning of the pandemic, the NIH, with inputs from academia and national experts, had drafted Pakistan’s National Action Plan for COVID-19 that outlined the key goals of the country’s response which grew iteratively with experience (17).

#### 3.2.1 Political challenges

The federal government was led by the political party that also controlled the government in Punjab and Khyber Pakhtunkhwa Azad Jammu and Kashmir and GB, while Sindh and Balochistan were led by other parties. Despite such consolidation of power, provinces, including those under the ruling party, pushed back sometimes including with nationwide political protests against the COVID-19 lockdowns (18). Provinces often did not share information with each other or the center, and there was little coordination between them. Differences also arose occasionally with ministries (e.g., for school closures or locking down highways) and with the private sector (e.g., holding or the scale of the Pakistan Cricket League, marriage ceremonies) (19, 20). Such disagreements were addressed in the NCC and the NCOC, through dialog, including accommodations such as delays in or toning down of lockdowns etc., while still keeping epidemic imperatives as the deciding yardsticks. The dialog approach helped develop consensus, collaboration and a more effective and coordinated pandemic response (21).

#### 3.2.2 Legal challenges

In March 2020, there was a legal vacuum to manage a national epidemic. The one federal law from the 1960s had become redundant due to subsequent legislations and evolution of the Constitution of Pakistan. The federal government could coordinate but had no legal mandate or authority to manage a national response to COVID-19. This context was addressed by creating new institutions of NCC and NCOC by the Federal Government through notification from the Prime Minister’s Office in March 2020. Effectively this redefined the *de facto* role of the federal government in the national response, without which, individual provincial responses risked being disparate, asynchronous, iniquitous, and possibly ineffective.

In the last quarter of 2020, once NCC and NCOC operations had become more established, the Islamabad High Court decided against a petition filed by private entities, questioning the legal authority of these institutions, and ordered that NCC/NCOC directions were binding on all provincial governments and citizens. The Supreme Court of Pakistan also ratified this position in a separate *suo moto* (on its own) review. Both these decisions helped consolidate the legal space for the federal government to manage the response. Also in the last quarter of 2020, the parliament passed a new legislation prepared by the ministry of health titled the “National Institutes of Health (NIH) Act,” creating a Center of Disease Control (CDC) at the NIH to address future health emergencies. However, allocation of adequate funding and personnel for the CDC/NIH remains a challenge to date.

## 4 Data use

The initial response to the pandemic included a review of existing processes and institutions to seek what could be coopted in the response.

### 4.1 Tracing, testing and quarantine (TTQ)

Before March 2020, Pakistan’s health surveillance system focused primarily on detecting vaccine-preventable diseases in children, particularly poliovirus, using approximately 1,400 case detection and verification teams located across the country. These teams were repurposed as “Rapid Response Teams (RRTs)” for the COVID-19 response, and additionally tasked with contact tracing, randomized sampling for disease prevalence in high-risk areas and school-based surveillance. Over time, the number of RRTs increased to 1,400 in Punjab (700 exclusively for COVID-19 and the rest with shared EPI responsibilities) and 700 each in all other provinces.

**Figure 2:**
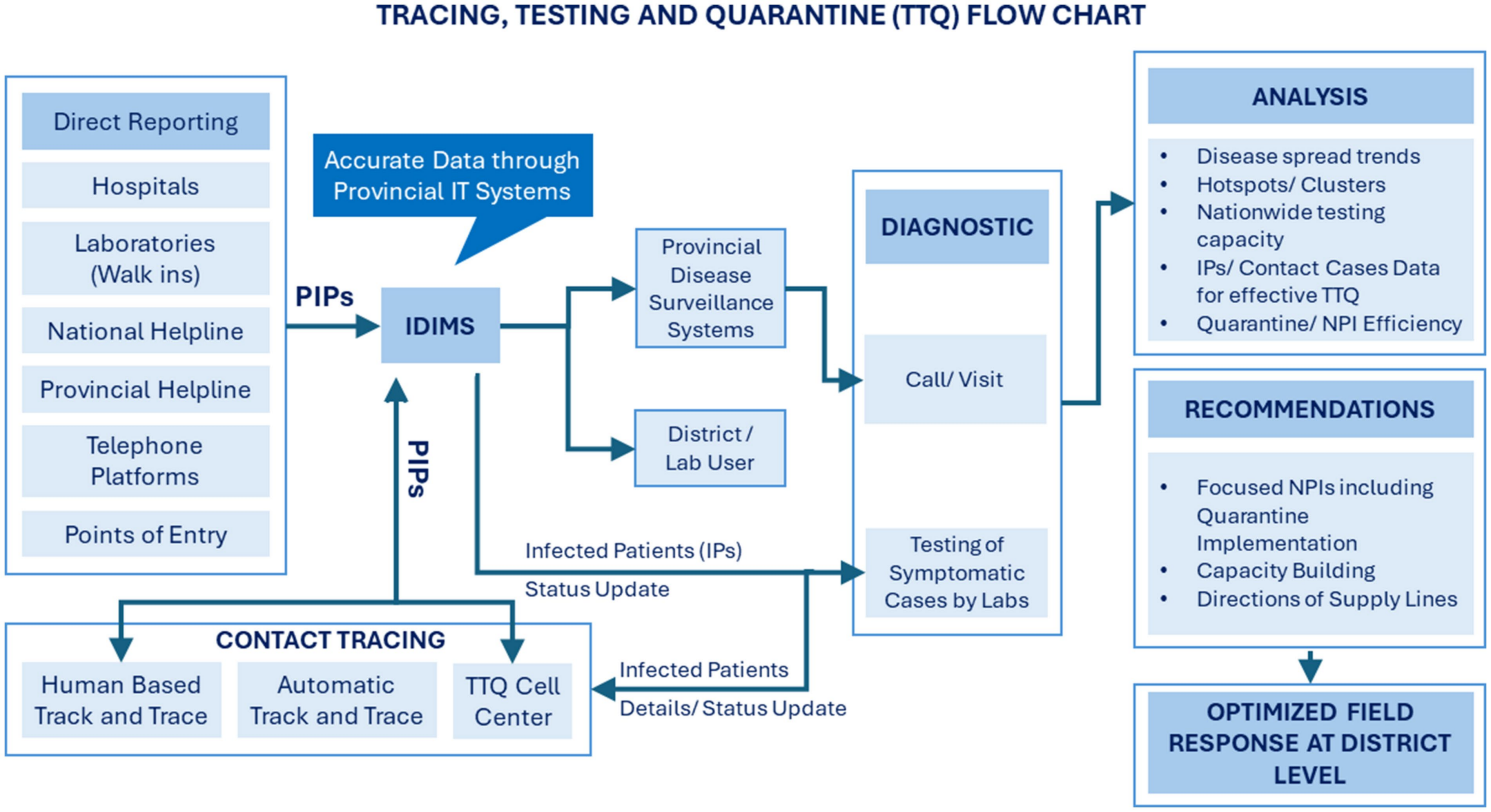
The setup of the tracing, testing and quarantine approach in Pakistan (13).

These allowed the institutionalization of a Tracing, Testing and Quarantine (TTQ) system in late April 2020 that was coordinated through a TTQ cell at the NCOC (Figure 2). The system collated all tests and contacts tracking, tests in educational facilities, data from hospitals and random testing in high-risk areas such as marketplaces, mosques, and shrines with large foot-traffic and thus high disease outbreak probability, data from testing of incoming travelers at airport and dry ports and testing data from rural areas, to track the spread of COVID-19.

The TTQ cell ensured effective collaboration for data between provinces, helped track disease spread, identified clusters/hotspots to enable smart lockdowns (by identifying high prevalence locations) and need-driven resource optimization at provincial, district, and tehsil levels. Data compiled by TTQ was part of the daily situation reports (DSRs) prepared by NIH which was the principal document on which the NCOC based its discussions and decisions.

### 4.2 Facility surveillance and case reports

Alongside case detection, a data sharing ecosystem was established. Initially, around 500 of the busiest major hospitals and laboratory networks throughout the country were included. This was expanded over time to include over 5,000 medium- to large-scale facilities. A pre-defined list of indicators was developed to capture key metrics, including cases, admissions, ventilator use, and deaths (22). This evolved over time as data needs were better understood. Although participation in the system was voluntary, an NCOC team initially assisted by local military units, followed up with facilities that did not enter data or encountered data quality issues. Some facilities reported later than others, yielding low case reporting over weekends followed by a surge in “catch up” reports by midweek. These variations were addressed by using weekly or three-day rolling averages. NCOC teams sometimes visited provinces/ large cities to address coordination issues as required.

An important piece in surveillance were the periodic reviews of burials in major city graveyards and local healthcare providers. NCOC teams would visit to ascertain the number of burials in a quarter compared to the same period the previous year. Similarly, teams would visit general practitioners in small towns to understand when there were fewer than anticipated cases from a locality. However, these were *ad hoc* activities and no formal records of these were created or retained.

### 4.3 Data management – types and conduits of data and its reporting

**Figure 3:**
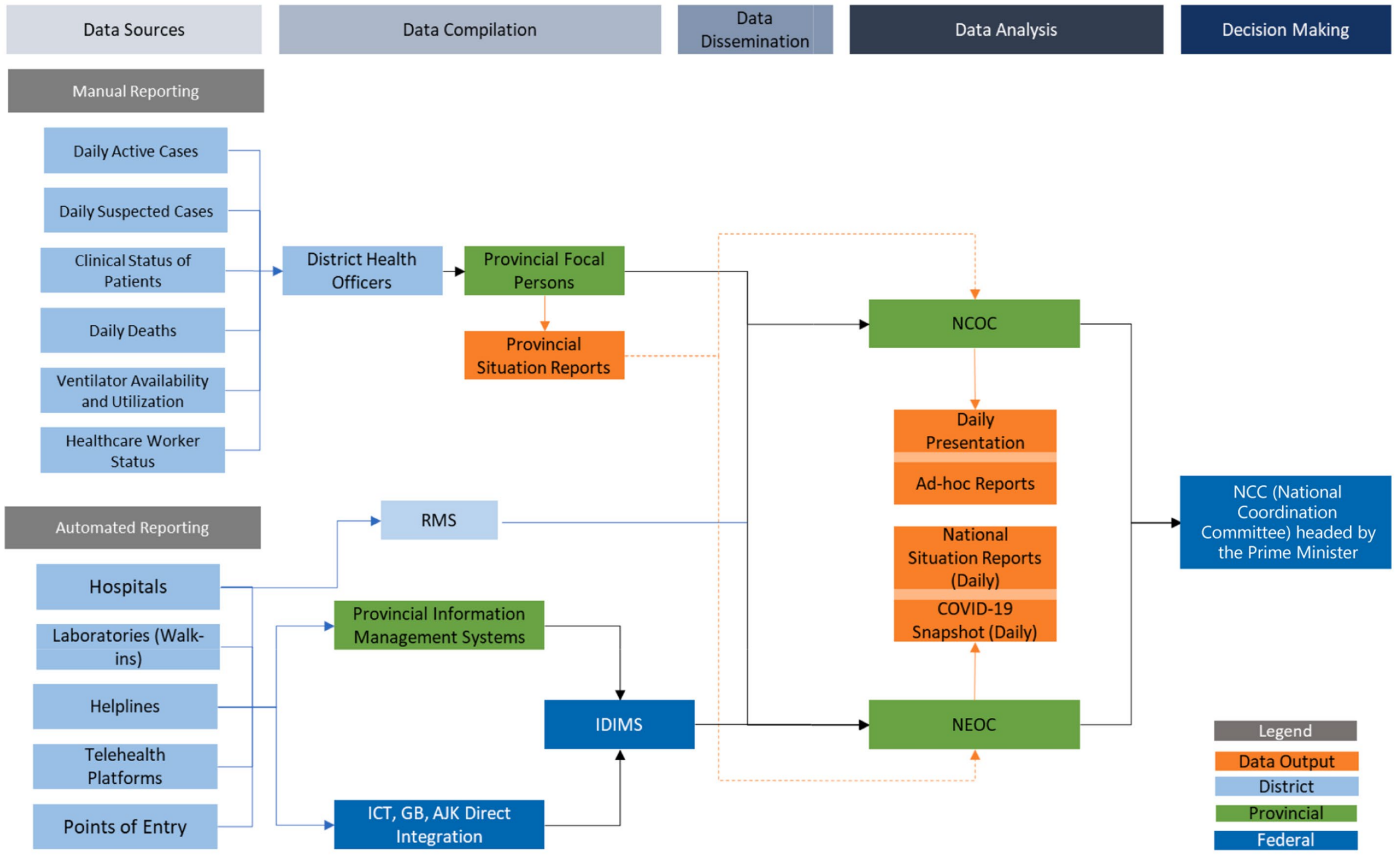
Data flowing into the NCOC for policy decisions.

The Expanded Program for Immunization (EPI) already had a sophisticated data management system to track data from field surveillance, vaccine logistics, and adverse effects from vaccines, that was housed at the National Emergency Operation Center (NEOC) for poliovirus control. This system was repurposed to manage additional COVID-19 surveillance data from both field and hospital cases. Data were entered manually by the many collection entities such as hospitals, laboratories, and district TTQ personnel. Once received, data were collated and cross referenced at the backend at the NEOC through automated protocols and application programming interfaces (API) (Figure 3).

Routines of data transmission were established. Data were collected at the district levels and transmitted electronically to provincial health departments between 8 and 9 pm daily, then on to the NEOC by midnight as Microsoft Word documents, PDF documents, and screenshots, where an IT specialist consolidated them into Microsoft Excel files, and also ran completeness checks before final collation. Collated data were analyzed using predetermined syntax and the outputs were presented to the Director General of MoNHSRC for verification and then shared with junior NCOC and MoNHSRC team personnel to prepare National Situation Reports (Sitreps). Sitreps were validated by senior NCOC officers before presentation at the 10 am daily meeting of the NCOC.

Samples were also collected from hospitalized patients and those presenting to specific sentinel laboratory sites in major cities and analyzed for virus variant types. National capacity was increased from scratch to testing of over 100 samples every month by early 2021.

### 4.4 Daily situation reports - DSR

The DSR prepared by NIH team contained daily updates of key population-level data, such as the number of daily tests (PCR and rapid antigen, separately), incident cases, COVID-19 positivity (%), deaths, recoveries, hospitalized and ventilated patients including among healthcare workers, hospital utilization, availability of oxygen beds and ventilators. New indicators, additional data and analysis were continuously added as needed to meet evolving needs for managing the epidemic. For example, disaggregation of data by cities and new cities were added over time. DSR also included as needed reports on national oxygen availability and capacity, drugs availability, key global and local trends, large upcoming events with superspreader potential and travel numbers, patterns, and issues both internally and from other countries.

### 4.5 Health information management systems

The NEOC upgraded the MoNHSRC’s Integrated Disease Information Management System (IDIMS) to incorporate COVID-19 data from hospitals (22). It also integrated with provincial data management systems to record cases, tests, deaths, hospitalizations, recoveries, and other pertinent indicators. The IDIMS database also contained the details of potentially infected persons (PIP) across the country. It was updated daily from laboratories all over Pakistan, through an online portal. The database contained individual level data about COVID-19 patients, including their name, gender, age, phone number, CNIC number, address, testing information, traveling history, and other clinical variables. There were some issues with late-reporting, inconsistencies, incompleteness, data-entry errors, and duplication that improved over time. Additionally, Punjab and Sindh maintained separate online reporting systems, albeit with inconsistent data. These systems ran parallel to the national system, which also included data from these provinces.

The NCOC, in collaboration with the National Information Technology Board (NITB, the government IT agency) created an online Resource Management System (RMS) to track resource availability at major hospitals across the country. It tracked availability of the number of beds, ventilators, patients on high-flow oxygen, and patients on low-flow oxygen. While hospitals connected to the RMS were expected to upload this data daily, a lag was often observed as data entry was voluntary by the hospital administration. Rapidity of its development left some functional errors, for example, the system to recorded beds and ventilators separately, resulting in a mis-approximation of hospital capacity^2^. The RMS received inputs from many sources, such as the Government of Pakistan’s App on COVID-19 management (i.e., Pak Nigehban), Education Information Management System (EIMS),^3^ the Federal Investigations Agency (FIA)‘s Passtrack App (for incoming international travelers),^4^ among others.

During the COVID-19 vaccination drive, a National Immunization Management System (NIMS) was developed in collaboration with the National Database and Registration Authority (NADRA), which issues national identification documents. NIMS was used for managing and monitoring citizen registrations, vaccines stocks, recording of data for administered doses and adverse events following immunization (AEFI). Supply chains at federal, provincial, and district stores, as well as health facilities administering the vaccine were managed using the COVID-19 Vaccine Inventory Management System (COVIM) developed by a private company, *Pace Technologies* (23).

The system was externally interfaced to allow citizen registrations through a SMS helpline number, upon which they received a text directing them to the nearest vaccination center where they could receive the vaccine on showing the SMS code they had received (24). Once they received the vaccine, their record was available immediately and could be retrieved online, through SMS or on a QR code readable card.

### 4.6 *Ad-hoc* analysis and the role of experts

Throughout the pandemic, the NCOC sought guidance from subject experts to inform their decisions to ensure evidence informed policies and implementation for the pandemic response. A committee of infectious disease specialists advised on disease management and later on about the vaccines, an eight-member medical expert team was initially organized by the Chinese government to provide consultations on pandemic control, patient treatment, and laboratory work, and to guide and train Pakistani medical staff and the World Health Organization (WHO) helped prepared Pakistan’s ‘Strategic Preparedness and Response Plan’ in the early days (25).

Routine data were compiled, and basic analysis were carried out by the NCOC team for daily Sitreps. A MoNHSRC embedded team from *Research and Development Solutions (RADS, funded by the Bill and Melinda Gates Foundation)* provided higher-level statistical analyses such as estimating and then measuring the impacts of school closures policy on COVID-19 transmission (26, 27), heatmaps to identify hotspots of COVID-19 cases, using Google Mobility data to monitor the implementation of NPIs, and assessing vaccine efficacy using population-level data. The team also conducted special surveys on vaccine acceptance and readiness (28–30), and tested experimental approaches to increase vaccinations in poor communities (22, 31).

The Health Planning Systems Strengthening and Information Analysis Unit (HPSIU), the Health Services Academy (HSA), RADS and the *Akhter Hameed Khan Foundation (AHKF)* conducted several *ad hoc* studies on aspects of COVID-19 attitudes (28), practices and challenges of healthcare providers, vaccine readiness and measured the impact of mitigation measures on maternal and child health. Specialized teams from *Love for Data (LFD)* developed an AI-based system to identify risk hotspots for COVID-19 using video-based risk detection (32). The *Institute of Health Metrics and Evaluation (IHME), HPSIU and LFD* provided forecasting of disease trends of COVID-19.

## 5 Non-pharmaceutical interventions enforcement

The Government of Pakistan implemented a range of non-pharmaceutical interventions to control the spread of COVID-19, that included mask-wearing in public, social distancing, lockdowns that ranged from nation or citywide to targeted smart and micro localized in individual localities, travel restrictions (both inbound international flights and inter-country travel), limitations on tourism, school closures, and restrictions on public gatherings and weddings (15, 26, 27, 33, 34). These measures, announced as Standard Operating Procedures (SOP), were implemented dynamically, with restrictions being eased and tightened based on real time information from individual localities.

**Figure 4:**
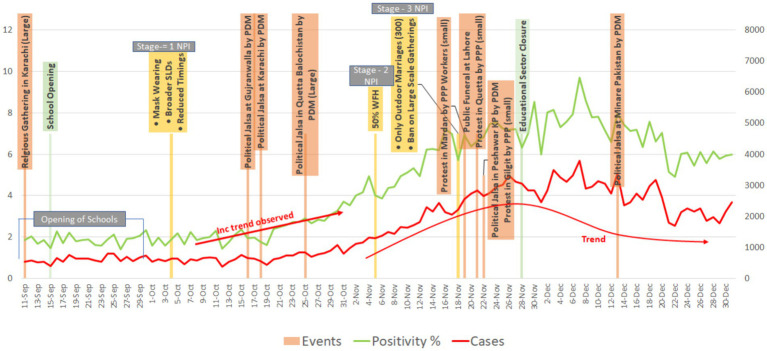
Effects of NPIs, political events and school closures on COVID-19 positivity (%) in 2020.

By late 2020, Pakistan’s containment measures had become well-established and effective (26), and were able to address subsequent waves of COVID-19 (Figure 4). As a result, government policy focus shifted from crisis management to enabling a return to normalcy with consequent changes in the national response. The first step toward this change was an overall easing of restrictions, particularly blanket lockdowns that had large economic and political costs. Based on analysis from analysis teams, a targeted approach was applied to monitoring and containing COVID-19 through smart lockdowns (SLDs) and mini-Smart lockdowns (mini-SLDs) at the street, neighborhood, or citywide levels. Predictions were made based on population density and local positivity. These locales were “sealed” by the local administration, restricting movement in and out of these areas. COVID-19 positivity rate was used as an indicator to monitor the spread of COVID-19 in these areas.

Throughout the latter months of 2020 and well into 2021, the targeted approach was applied whenever case positivity rose (on tests from hospitals, laboratories and TTQ), institutionalization of SLDs or mini-SLDs, periodic school closures, lowered attendance at wedding or other public functions, reducing market hours, and limiting public gatherings. This approach continued irrespective of the COVID-19 variant that was driving that wave.

The quality of implementation was monitored daily by TTQ and special NEOC/PEOC teams that reported with pictures, and rapid surveys to assess mask wearing, number of people in marketplaces, and attendances at public events such as wedding or religious ceremonies. Occasionally, artificial intelligence-based algorithms were applied to quantify this effectiveness of implementation, for example by estimating the percentage of masking in public places. However, this was sporadic given its high costs. Initially many SOP were followed voluntarily. However, as vaccination rates grew and people felt more comfortable, adherence fell. Since ensuring compliance with such SOP were resource intensive, by 2022, as a significant proportion of the population had been vaccinated, most restrictions started being lifted.

## 6 Support responses – engagement with ministries and the private sector

A core feature of NCOC was its intersectoral and inter-ministerial approach to formulate a unified COVID-19 pandemic response. The NCOC collaborated with various government ministries, civil administration, private sector organizations, and civil society organizations to develop and implement strategies to contain the spread of the virus. Daily meetings at the NCOC helped with the coordination. Various ministries played crucial roles. For example, the Ministry of Interior was crucial in enforcing lockdowns and implementing SOP across the country (28, 29). The Ministry of Industries and the National Disaster Management Authority (NDMA) led the ramp up in the capacity to meet oxygen needs in hospitals (35).

As hospitalizations rose, there was a critical shortage of medical oxygen. The national supply at the time was around 350 metric tons a day of which two thirds was for commercial or industrial use. At the peak of hospitalizations, particularly during the third wave (Table 1), medical oxygen requirements exceeded 400 MT/D. As a result, the bulk of the oxygen produced in the country was diverted toward the treatment of COVID-19 patients, exacerbating the scarcity of medical oxygen for other medical conditions (34). In the short-term oxygen was diverted from industry to medical use. In the longer time, local oxygen generation capacity was increased. For example, the Chinese government donated 200 oxygen generators (concentrators) worth 3 million yuan ($463,943) (36), and the United Nations Children’s Fund (UNICEF) contributed 1,000 oxygen concentrators and related accessories valued at USD 1.4 million (37). Other friendly countries such as Saudi Arabia also funded to build further capacity, to the point that the national capacity had increased to over 800 MT/D toward the end of the fifth wave. A new critical care hospital was established at NIH, Islamabad co-funded by Government of Pakistan and China. The prefabricated building was completed by NDMA in less than 2 months and its personnel were hired by the health ministry through walk-in interviews in days through a unique arrangement of service agreements with the approval from the federal cabinet.

Other collaborations included the Federal Ministry of Education, provincial education, and private schools’ organizations for policy decisions regarding closing or reopening educational institutions to control disease spread. The Ministry of Religious Affairs, the Pakistan Ulema Council (PUC), a nongovernment organization comprising of Islamic scholars from different school of thought and the Council of Islamic Ideology (CII), a federal constitutional body that provides advice on Islamic law, also issued guidelines and decrees to promote the postponement of religious gatherings and adherence to government SOP.

Charitable organizations such as the Chhipa Welfare Association and the Edhi Foundation provided relief packages and essential goods to thousands of poor households affected by the pandemic, along with ambulance services for COVID-19 patients (38). A number of philanthropies supported individuals that faced economic hardship due to limitations in employment during lockdowns. A large rural focused consortium of NGOs, the Rural Support Programs (RSPs), were engaged repeatedly to implement SOP to support vaccination and to identify poor households for distribution of food and other necessities (39).

## 7 Vaccination

### 7.1 Institutional setup

Preparations for acquiring and administering COVID-19 vaccinations began in July 2020 and included direct oversight from the Prime Minister by forming the National Vaccine Task Force (NVTF) to provide oversight. The NVTF included staff from various federal ministries and a committee of national experts to provide up-to-date technical and scientific advice on COVID-19 vaccines (Figure 4).

A novel arrangement was put in place by the Ministry of Health to manage the vaccine procurement, which was just not possible under the government’s Public Procurement Regulatory Authority (PPRA). The federal cabinet approved the establishment of a Cabinet Committee after receiving approval from the PPRA Board to dispense with PPRA law and regulations for procurement of vaccine. This committee had the authority to make all necessary decisions for vaccine procurement. It could make such decisions without repeatedly returning to the federal cabinet for further approval, as specified under government rules for powers devolved to provinces. This arrangement provided MoNHSRC and NDMA with the necessary protection and expedited timely decisions. A major decision by the cabinet committee, prompted by MoNHSRC, was to procure vaccine directly from the manufacturers without involving any middleman, thus enhancing transparency of the procurement process.

**Figure 5:**
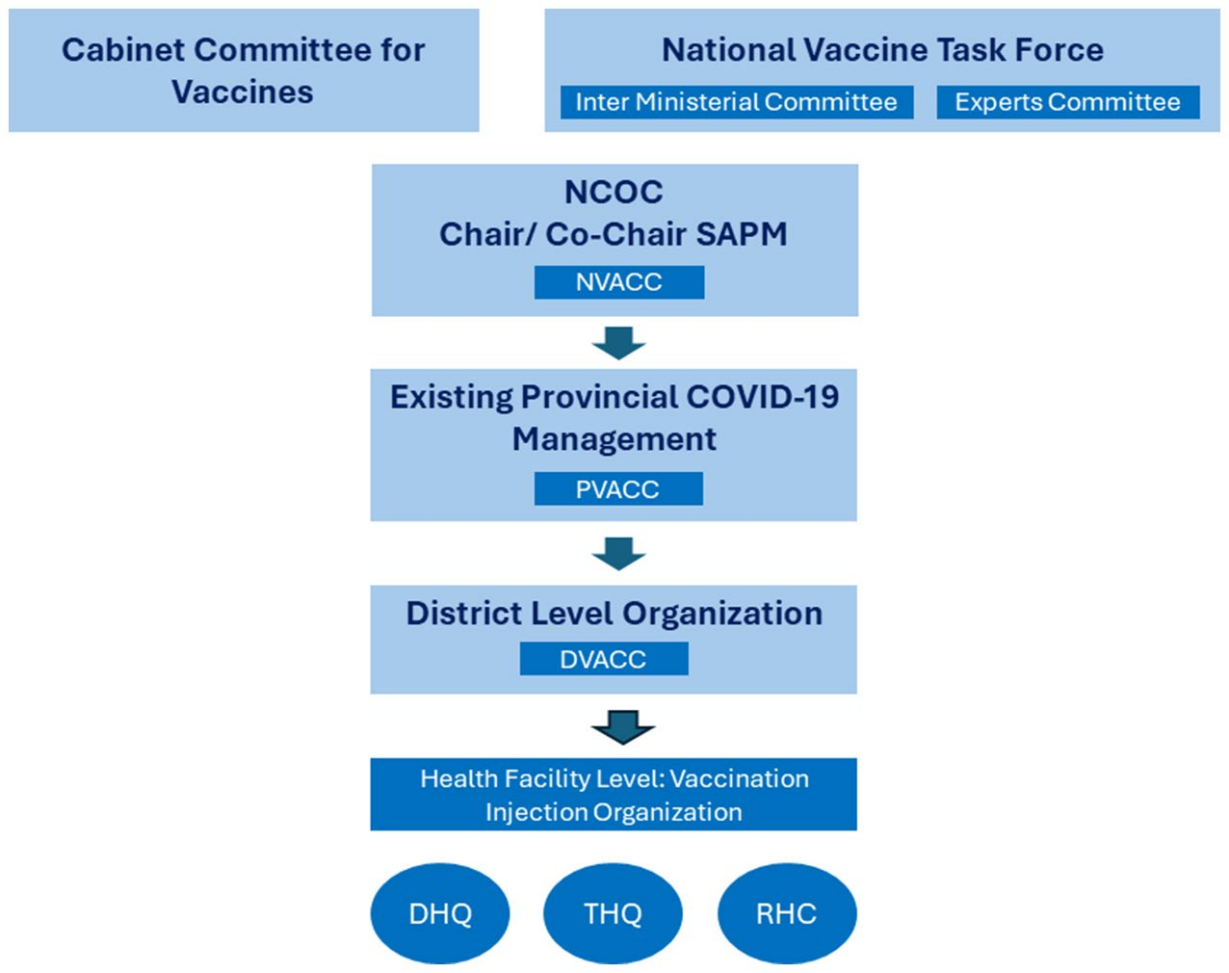
Governance structure for vaccine plan.

A National Vaccine Administration and Coordination Cell (NVACC) was established within the NCOC to operationalize policy directives from the NVTF and Cabinet Committee, working in coordination with the MoNHSRC (Figure 5). NVACC helped refine and review the national vaccine strategy, oversaw operationalization and taking remedial actions as needed, facilitated vaccine procurement and the vaccine supply chain (including arranging vaccine cold storage), and coordinated vaccine-related communication by the Government. It leveraged existing vaccination infrastructure of the EPI for the vaccine rollout. It fostered creation of guidelines and training vaccinators along with setting up IT systems to collect, collate, manage, and analyze vaccine-related data coordinated with the Provincial Vaccine Administration and Coordination Cells (PVACCs) to perform these tasks.

The MoNHSRC/EPI liaised with the COVAX facility of the Gavi to procure vaccine, transport, and to arrange for cold storage for the acquired vaccines. EPI mainly played a role in logistics of the vaccines, while actual vaccinations were administered through the NVACC and provincial health and civil governments.

Multilateral and bilateral development partners supported vaccine procurements and other functions. They operated through the Development Partner Coordination Committee (DPCC), which liaised with the federal EPI to secure essential vaccines and related finances and supported provincial governments to establish on-ground managerial mechanisms for the vaccine rollout.

The Drug Regulatory Authority of Pakistan (DRAP) played a crucial role in taking prompt decisions and providing legal oversight and regulation of vaccines. DRAP approves drugs for use in Pakistan, including in emergencies such as the COVID-19 pandemic. The drug regulator receives detailed human trial data from vaccine manufacturers before it approves any vaccine for use in Pakistan.

### 7.2 Vaccine supply

In the beginning, supply of vaccine was limited worldwide, while new vaccines were coming on board and their regulatory approvals were becoming available practically in real-time. DRAP and the experts committee actively reviewed documents as they became available and made rapid decisions. Beyond choosing what to buy, the availability was a challenge. In early 2021, countries which were large producers of vaccine such as United States of America and India placed export bans to prioritize vaccination of their own populations. This left a much of the world without vaccines in the first half of 2021. For example, in early March 2021, over one million doses of AstraZeneca vaccines were to arrive with support from Gavi but were delayed and then later canceled as the manufacturer was unable to meet pledged demand. Ultimately first doses of the Sinopharm^®^ vaccine (by the China National Pharmaceutical Group) became available, first as a gift, then on subsidy from the Chinese government and then on full payment in sufficient quantities to start the national vaccination program. The national vaccination campaign was made possible from February 2021 with vaccine supplied through the highest-level intergovernmental dialog, where the Chinese Government agreed to divert some of its supplies to Pakistan despite its resolve to vaccinate 100% of its own population by June 2021. Vaccines from COVAX, the COVID-19 Vaccines Global Access coalition, became available somewhat later (40).

The government also signed an agreement with the manufacturer CanSinoBio in March 2021 to import bulk vaccine concentrate of SinoVac vaccine to start co-production and repackaging of the vaccine in Pakistan as PakVac (40). Thirty million vaccines were produced under this arrangement at NIH. Being a single-use vaccine, it played a crucial role in providing vaccination cover to the population quickly. Over time, other vaccines became available, particularly from COVAX. These included the BioNTech/ Pfizer vaccine, which became the predominant vaccine by fall of 2021.

### 7.3 Vaccination rollout – the early preparations

The nationwide vaccination effort required several preparations. A COVID-19 vaccine supply information management system COVIM (above) was developed in collaboration with the EPI. NADRA and NCOC developed a vaccination tracking system (NIMS, above) where individuals could register, receive instructions, and track their vaccination status. The system also allowed an outsider, for example an airport official anywhere in the world, to verify someone’s vaccination status through a QR code. These systems were interfaced with API to ensure daily data reporting in real time that NCOC used to make daily operational decisions by location across Pakistan.

National and provincial health authorities reconfigured several existing health facilities into COVID-19 vaccination centers nationwide. This was simpler initially when many outpatient departments were closed due to COVID-19, and their space could be re-purposed. However, as outpatient services resumed, the influx of those seeking vaccinations became overwhelming and centers were moved to public spaces such as parks and other public buildings. A key problem that arose was that many of the centers were too far away from some of the poorest citizens (30). This was addressed by sending mobile vans into urban informal settlements and other poor communities. However, initially these were met with low turnout and only increased once local organizations were involved in preparing and supporting van visits (31).

### 7.4 Vaccination rollout – prioritizing populations

Given the supply constraints, NCOC prioritized vaccinations initially for the older adult (80 years and older initially), healthcare workers, and those at risk due to specific medical conditions. The age for vaccination was gradually lowered as vaccine supply improved in the last half of 2021, allowing equitable and effective vaccination of the population based on those in greatest need of the protection (39). Vaccination became available to everyone 18 years or older by June 2021.

Due to a lack of safety data, sub-populations such as pregnant women and individuals under the age of 18 years were initially excluded from receiving the vaccine. Based on internal analysis of cases and probability for infections, urban populations were prioritized. After these exclusions it was estimated that around 100 million individuals would need to be vaccinated, and initial benchmark of reaching 70% of this population or 70 million was set for the end of 2021. To achieve this target, the government allocated PKR 1.1 billion in the Pakistan Federal Budget 2021–22 (40, 41).

By September 2021, the eligibility was expanded to include pregnant women and children above 12 years based on newer safety data (42). Given the rapidity of the rollout, rural areas were also targeted. By then, several millions of doses of mRNA vaccine had been received from the United States Government, along with a steady supply of the Chinese vaccines, which made it possible to include all eligible population.

For most part Pakistan’s vaccination program progressed rapidly, and most targets were met early. There were occasional slowdowns, particularly as saturation was approached and, issues of vaccine hesitancy, social challenges, reaching marginalized populations, and logistics in remote areas became more prominent. However, they were managed through meticulous daily/ weekly planning at the NCOC and follow up of progress in real time at the sub-district levels.

## 8 Communications and information

Effective communication was a cornerstone of Pakistan’s COVID-19 response. A *Communication Cell* was created at the Ministry of Health with the assistance of Bill and Melinda Gate Foundation and UNICEF, where a team of communication experts guided messaging, and gaged, monitored and responded to any misinformation or disinformation on all aspects of COVID spread and evolution of the disease, and about vaccine or epidemic countermeasures on social media, print media and electronic media. It also helped coordinate among government departments, disseminating COVID-19 guidelines to a diverse set of public and private stakeholders, and keeping the public up to date on cases, preventive measures, and vaccination. Under guidance of the health ministry, the cell gave daily or weekly briefings to the NCOC and monthly reports to the ministry on all issues and steps taken to counter false or incorrect information.

The NCOC convened various private stakeholders and government departments at federal or provincial levels to build consensus and chalk out strategies. Communication at the NCOC was guided by the MoNHSRC and operationalized by the Ministry of Information and Broadcasting and the military’s Inter Services Public Relations (ISPR) wing. Relevant content was produced regularly, sometimes daily and communicated through various channels, including conventional mail, telephone, print media, press releases, official notifications, infographics, SMS, public service message videos, TV and radio shows, digital and social media, caller ring tones, and a dedicated website ^5^that depicted daily changes in the epidemic situation and vaccination status. Explanations were provided to help the public understand the situation, so that terms such COVID-19, virus, positivity, etc. became part of daily vernacular. Important events and news were conveyed directly from NCOC through press conferences held by the ministers of health and planning and the DG NCOC.

Technology guided the vaccination rollout. People were reached and could check their eligibility status, find vaccination centers, and book vaccination appointments through mobile phones. A dedicated portal was created by the NADRA for the public to retrieve vaccination records and certificates. The front-end access was available through website ^6^and the PAK COVID-19 Vaccination Pass application was deployed on Android and Apple platforms.

## 9 Funding

Pakistan funded the response to the COVID-19 pandemic from multiple sources. Initial allocation was for PKR 1.240 trillion through the Economics Stimulus Package (ESP) (43). By June 30, 2020, PKR 354.23 billion (USD 2.25 billion), had already been released to address aspects of the response. An Emergency Response Package was established within the ESP for PKR 190 billion, to support the NDMA, provide medical equipment and incentives for healthcare workers, offer emergency relief funds (including cash relief through the Benazir Income Support Program), and grant tax relief on essential food and health items. Furthermore, an Emergency Relief Fund was established by the Finance Division, with an additional grant of PKR 417.28 billion. The fund included PKR 380 billion from a supplementary grant and PKR 100 billion from the Residual/Emergency Relief Fund of the ESP to eventually total PKR 480 billion for emergency relief efforts.

In addition to the government funding initiatives, Pakistan received substantial grants and loans from international donors to support its response to the COVID-19 pandemic. Multilateral grants totaling USD 28.45 million were committed by the Asian Development Bank (ADB), European Union (EU), Islamic Development Bank, and the United Nations (UN). Bilateral grants amounting to USD 70.626 million were also pledged by countries including Canada, China, Japan, South Korea, the United Kingdom, and the United States. In addition, ADB, Islamic Development Bank, and the World Bank committed loans worth USD 721.17 million (43).

## 10 Transition to post-emergency setup to NIH

In Pakistan, the National Institute of Health (NIH) is responsible for managing epidemics and infectious diseases as it serves as the surveillance and implementation arm of the MoNHSRC. However, when COVID-19 pandemic started, limited capacity at the NIH necessitated the formation of the NCOC to control the pandemic. After successfully managing the national response, the NCOC was dissolved in March 2022. The administration of vaccines and monitoring of pandemics were then officially transferred to the NIH, which took over surveillance responsibilities at the end of the fifth wave of the pandemic, albeit at the pre-epidemic levels (44).

## 11 Discussion and lessons learnt

Pakistan had many of the elements needed to mount an effective response to the COVID-19 pandemic in 2020. However, several of these existed in silos, with little mutual coordination and performed inconsistently. The crucial driver of Pakistan’s very effective response was the establishment of a coordination system that was led by the top leadership in Pakistan, and included various political, military, and civil society actors in a common decision making and implementation mechanism. This coordination was top down, yet inclusive of disparate views, respectful of provincial and private sector autonomies, yet decisive and single minded when difficult decisions were needed. It built or repurposed the data and implementation systems that were needed and relied heavily and routinely on data to hold implementers accountable.

Pakistan has a very large healthcare infrastructure that includes more than 18,000 public sector and likely over 75,000 private health facilities, and disease specific surveillance for polio and measles. Data systems in the private sector seldom feed into any national repository. Most importantly, the NIH where the COVID-19 response could have been housed was insufficiently staffed or motivated to participate, leaving a vacuum that had to be filled. Finally, since the 18th Amendment to the Constitution in 2010, provinces have felt strongly about their autonomy and seldom communicate or collaborate with each other. The NCOC was able to provide that coordination platform. This support was widely accepted by provincial governments, civic organizations, and the society at large partly due to the unprecedented situation unfolding globally and a broad-based national consensus on the necessity for an effective response to the pandemic. Provinces discussed and addressed differences. Decisions followed data, to ensure ideas were translated into practice and identify lags in implementation and fixed it through troubleshooting (7, 9, 10). Overall, at least part of the consensus came from the sense that the NCOC was making data-based decisions, that were fair and that the quality and scope of data brought into use was superior to anything provincial officials had access to on their own.

The political arrangement of the COVID response was crucial. All federal minsters participated at the NCC where provincial chief ministers were also invited regularly. The NCOC leadership included the planning minister to facilitate financial allocations, the health minister provided technical expertise and the military team ensured implementation in two ways. Firstly, it provided personnel to carry out tasks. At its peak, NCOC had nearly 300 personnel assigned to it, including two one- or two-star generals and over 10 brigadiers. By contrast, most ministries do not have nearly that many personnel. For example, the MoNHSRC has 31 sanctioned officer positions at its head office, and fewer than 5 of these are grades 20 or above, and only around a third are filled at any point. Having sufficient personnel and of appropriate seniority and experience was crucial, as those assigned to the NCOC could dedicate all their time to NCOC work, whereas most MoNHSRC and NIH personnel would have split their time between COVID-19 work and their routine assignments simultaneously. Having additional personnel meant that military staff could be assigned as additional personnel when needed in districts. Finally, having uniformed staff provided necessary impetus as teams could induce work from district or even provincial administrations.

One of the most important lessons learnt was that the senior leadership in Pakistan saw COVID-19 as an existential and political threat and responded accordingly. All actors came aboard, although there were differences, that were aired and addressed, and definitive actions were taken. Even while the response was ongoing, the level of implementation, cooperation between actors, and compliance by the population waxed and waned, in time with the ebb and flow of the epidemic. However, once survival pressure abated, the situation reverted to the usual patterns with lowered focus on results or coordination (45).

Success of compliance of the public with the restrictions placed during the response partly reflects the authoritarian nature of the implementation process. COVID-19 SOP were communicated to the public as directives, and violations by businesses or individuals were penalized (1, 46, 47). This likely contributed to the overall effectiveness of the SOP enforcement in Pakistan. Globally, such authoritarian approaches were part of many successful responses (10, 48), and reflect the Ding and Li framework of “rough rationality with limited resources” (49). In essence there is a tradeoff between using limited resources to find and heavily penalizing a few offenders publicly vs. universal enforcement, testing and contact tracing (50). Pakistan opted for the former given its resource constraints (11, 51).

## 12 Conclusion

The experience of managing the COVID-19 response successfully created many lessons that can inform a more proactive approach to emerging health and other threats, particularly in resource constrained countries (9). These lessons inform about diverse aspects of resource allocations, institutional arrangements, galvanizing the civil society and general public, and generating political consensus. These unique lessons allow insights into how a socio-economically challenged country with a wide array of constitutional and political actors with diverse ethnicities worked toward reducing the impact of COVID-19 despite their often-diverging priorities. Of importance is how although there were several government entities that are tasked with aspects of such responses, seldom talked with each other, or used data to guide or coordinate actions (52). This status quo reflected, and still does in the post COVID-19 times, of political economy of government functions that are conducted as “activities” rather than to achieve policy objectives such as managing current or mitigating future emergencies. They were brought together on an effective platform that measured and delivered results when an imminent and existential threat was perceived. However, there is a danger that the many lessons learned from responding to COVID-19 may be lost now that the existential threat has receded (45).

## Data availability statement

The data analyzed in this study is subject to the following licenses/restrictions: the data was provided to the Akhter Hameed Khan Foundation team for this study as part of its work with Pakistan’s Federal Ministry of National Health Services, Regulations & Coordination (MoNHSRC) and the National Command & Operation Centre (NCOC) in Islamabad, which lead Pakistan’s response to the COVID-19 pandemic. The AHKF team has provided analytical support to the above entities, and such created knowledge that has directly informed pandemic policy-making in Pakistan. COVID-19 data is compiled and shared in daily National Situation Reports, or Sitreps, by the National Emergency Operation Centre (NEOC). The parentage of this data is with the NCOC and the MoNHSR&C. The AHKF team received this data with the express understanding that it would be kept confidential. However, the data can be obtained independently from the NEOC, through a data request procedure, which is subject to approval from the MoNHSRC. Requests to access these datasets should be directed to Dr. Shahzad Baig, Email: eocpakistan@gmail.com.

## Author contributions

AdK: Writing – review & editing, Validation, Supervision, Project administration, Methodology, Investigation, Funding acquisition, Formal analysis, Data curation, Conceptualization. MA: Writing – review & editing, Writing – original draft, Resources, Methodology, Investigation, Formal analysis, Data curation, Conceptualization. RK: Writing – review & editing, Writing – original draft, Resources, Methodology, Formal analysis, Data curation. TK: Writing – review & editing, Writing – original draft, Resources, Investigation. FS: Writing – review & editing, Validation, Supervision, Resources, Project administration. SA: Writing – review & editing, Resources. AaK: Writing – review & editing, Validation, Supervision, Resources, Project administration. AyK: Writing – review & editing, Validation, Supervision, Project administration, Methodology, Funding acquisition.

## Footnotes

^1^Using a graphical representation of the epi-curve of daily new COVID-19 cases, the day with the lowest number of daily new COVID-19 cases before cases began to steadily rise, never dropping below x number of cases, was taken as the start of a wave. The end date was the day with the lowest number of cases (y), toward the winding down of a wave, such that the number of cases on each of the 7 days before the day and on each of the 7 days after the day were higher than y number of cases.

^2^For a critical care COVID-19 patient bed and ventilator are a joint requirement, this means that in certain cases while the RMS shows the availability of a bed the required ventilator is not present, thus a patient cannot be admitted.

^3^Recorded information on public/private educational institutes (number of students, staff etc.).

^4^Application records information of individuals flying in from other countries and their COVID-19 status.

^5^ covid.gov.pk

^6^ nims.nadra.gov.pk
